# Loss of structural balance in stock markets

**DOI:** 10.1038/s41598-021-91266-4

**Published:** 2021-06-09

**Authors:** Eva Ferreira, Susan Orbe, Jone Ascorbebeitia, Brais Álvarez Pereira, Ernesto Estrada

**Affiliations:** 1 Department of Quantitative Methods, University of the Basque Country UPV/EHU, Avda. Lehendakari Aguirre 83, 48015 Bilbao, Spain; 2 Department of Economic Analysis, University of the Basque Country UPV/EHU, Avda. Lehendakari Aguirre 83, 48015 Bilbao, Spain; 3 Nova School of Business and Economics (Nova SBE) NOVAFRICA, and BELAB, Carcavelos, Portugal; 4 Institute of Mathematics and Applications, University of Zaragoza, Pedro Cerbuna 12, 50009 Zaragoza, Spain; 5 ARAID Foundation, Government of Aragon, Zaragoza, Spain; 6 Institute for Cross-Disciplinary Physics and Complex Systems (IFISC, UIB-CSIC) Campus Universitat de les Illes Balears, 07122 Palma de Mallorca, Spain

**Keywords:** Complex networks, Applied mathematics

## Abstract

We use rank correlations as distance functions to establish the interconnectivity between stock returns, building weighted signed networks for the stocks of seven European countries, the US and Japan. We establish the theoretical relationship between the level of balance in a network and stock predictability, studying its evolution from 2005 to the third quarter of 2020. We find a clear balance–unbalance transition for six of the nine countries, following the August 2011 Black Monday in the US, when the Economic Policy Uncertainty index for this country reached its highest monthly level before the COVID-19 crisis. This sudden loss of balance is mainly caused by a reorganization of the market networks triggered by a group of low capitalization stocks belonging to the non-financial sector. After the transition, the stocks of companies in these groups become all negatively correlated between them and with most of the rest of the stocks in the market. The implied change in the network topology is directly related to a decrease in stock predictability, a finding with novel important implications for asset allocation and portfolio hedging strategies.

## Introduction

The complex nature of the stock market is condensed in the phrase: “Economists are as perplexed as anyone by the behavior of the stock market”^1^. Understanding this complex behavior—characterized by a combination of saw-tooth movements and low-frequency upward and downward switches^2^—is a major goal in finance. However, the importance of stock markets goes beyond finance to impact macroeconomic modeling and policy discussion^3^. In fact, it has been recognized that the stock market is a good predictor of the business cycle and of the components of gross national product of a given country^3^. Special emphasis has been placed on understanding the role of risk and uncertainty in stock markets from different perspectives^4–16^, with a focus on security pricing and corporate investment decisions. At the end of the day, as said by Fischer and Merton^3^: “in the absence of uncertainty, much of what is interesting in finance disappears”.

As the majority of complex systems, stock markets have an exoskeleton formed by entities and complex interactions, which give rise to the observed patterns of the market behavior^17–20^ (see Ref.^21^ for a review of the recent research in financial networks and their practical applicability). For the same set of entities, there are different forms to define the connectivity between them. A typical way of connecting financial institutions is by means of borrowing/lending relations^21–24^. However, in the case of stock markets, where stocks represent the nodes of the network, the inter-stock connectivity is intended to capture the mutual trends of stocks over given periods of time. Mantegna^25^ proposed to quantify the degree of similarity between the synchronous time evolution of a pair of stock prices by the correlation coefficient $\rho _{ij}$ between the two stocks *i* and *j*. Then, this correlation coefficient is transformed into a distance using: $d_{ij}=\sqrt{2\left( 1-\rho _{ij}\right) }$. Mantegna’s approach has been widely extended in econophysics^26^ where it is ubiquitous nowadays as a way to define the connectivity between stocks. This approach has been used for instance for the construction of networks to analyze national or international stock markets^27–32^. A characteristic feature of this approach is that although $\rho _{ij}\in \left[ -1,1\right]$, the distance $d_{ij}$ used as a weight of the links between stocks is nonnegative. This allows the use of classical network techniques for their analysis (see Ref.^33^ for a review). To avoid certain loss of information inherent to the previous approach^34^, Tse et al.^35^ decide that an edge exists between a pair of stocks only if $\rho _{ij}>\left| z\right|$ for a given threshold *z*, which however retains the nonnegativity of the edge weights.

The importance of allowing explicit distinction between positive and negative interdependencies of stocks, which is lost in the previous approaches, was recently remarked by Stavroglou et al.^36^. They built separated networks based on positive and negative interdependencies. This separation may still hide some important structural characteristics of the systems under analysis. Fortunately, there is an area of graph theory that allows studying networks with the simultaneous presence of positive and negative links. These graphs, known as signed graphs, are known since the end of the 1950’s^37^ (see Ref.^38^ for a review), but only recently have emerged as an important tool in network theory^39–43^. A focus on signed networks has been the theory of social balance^37,44^. It basically states that social triads where the three edges are positive (all-friends) or where two are negative and one is positive—the enemy of my enemy should be my friend—are more abundant in social systems than those having all-negative, or two positive and one negative ones. The first kind of networks are known as balanced, and the second ones as unbalanced (see Refs.^39–43^ for applications).

Here we introduce the use of signed networks to represent stock markets of nine developed countries and to study the degree of balance. First note that in finance, the time varying behavior of networks is an important characteristic, since the time varying comovements become a risk factor^45^. Hence, we analyze the time variation of the degree of balance on stock networks between January 2005 and September 2020, thus addressing a gap pointed out in a recent study^21^. To build the graphs we use the rank correlations between stocks, which provide a measure of similarity better suited for financial distributions. We find a balance-unbalance transition (BUT) in six of the countries studied. The BUTs occurred around September/October 2011 for the US, Greece, Portugal, Ireland, and Spain, and later in France, which took place just after the Black Monday in August 2011. Neither Germany, nor Italy, nor Japan showed clear signs of this BUT. We discover that the observed BUTs are mainly triggered by a reorganization of the topology of the stock market networks. They consist of the movement of a few low capitalization stocks from the periphery to the center of the networks by forming cliques of fully-negative interdependencies among them and with most of the rest of the stocks in the market. These transitions impact directly on the naive predictability of stock prices from pairwise rank correlations. This is a novel finding with a direct impact on optimal asset allocation and hedging, which opens promising avenues for future research.

## Theoretical approaches

### Correlations and predictability of interrelated stock prices

To study the similarity between stock price changes we consider the time series of the log returns of stocks, $Y_{i}\left( t\right) =\log \left[ P_{i}\left( t\right) /P_{i}\left( t-1\right) \right] ,$ where $P_{i}\left( t\right)$ is the daily adjusted closing price of the stock *i* at time *t* for $t=1\ldots S$. Let us consider the log return of three stocks forming the vectors $Y_{1}$, $Y_{2}$ and $Y_{3},$ such that $Y_{i}=\left[ Y_{i}\left( 1\right) ,\dots ,Y_{i}\left( S\right) \right] ^{T}.$ The similarity between the orderings of the log returns $Y_A$ and $Y_B$ of two stocks *A* and *B* can be captured by the Kendall’s tau

1 $$\begin{aligned} \tau =2{\mathcal {P}}\left( \left( Y_{A}-Y'_{A}\right) \left( Y_{B}-Y'_{B}\right) >0\right) -1, \end{aligned}$$

where $Y_{\ell }'$ is an independent copy of the vector $Y_{\ell }$, $\ell =A,B,$ and ${\mathcal {P}}(X)$ is the probability of the event *X*. If two rankings are concordant, $\tau >0$, if two rankings are independent, $\tau =0$, and if the two rankings are discordant, $\tau <0$.

When considering the correlations between three stocks forming a triad there are four cases that can emerge: (i) the three pairs of stocks are correlated: $\tau \left( Y_{1},Y_{2}\right) >0$, $\tau \left( Y_{1},Y_{3}\right) >0$ and $\tau \left( Y_{2},Y_{3}\right) >0$; (ii) one pair of stocks is correlated and two pairs are anticorrelated: $\tau \left( Y_{1},Y_{2}\right) >0$, $\tau \left( Y_{1},Y_{3}\right) <0$ and $\tau \left( Y_{2},Y_{3}\right) <0$; (iii) two pairs of stocks are correlated and one pair is anticorrelated: $\tau \left( Y_{1},Y_{2}\right) >0$, $\tau \left( Y_{1},Y_{3}\right) >0$ and $\tau \left( Y_{2},Y_{3}\right) <0$; (iv) the three pairs of stocks are anticorrelated: $\tau \left( Y_{1},Y_{2}\right) <0$, $\tau \left( Y_{1},Y_{3}\right) <0$ and $\tau \left( Y_{2},Y_{3}\right) <0$.

Let us focus on estimating the trend of one of the stocks from another in the correlation triad considering a time varying regression model (see Appendix B in the Supplementary Information (SI)). Let us write ${\hat{Y}}_{i}(t)\left( Y_{j\ne i}(t)\right)$ for the nonparametric smoothed estimate of $Y_{i}$ from $Y_{j}$: ${\hat{Y}}_{i}(t)={\hat{m}}_{i}(Y_{j}(t))$, where $m_{i}(\cdot )$ is a non-specified unknown function. Then, for any stock, e.g., $Y_{1}$, we can make two predictions from another stock, e.g., $Y_{2}.$ One of them is simply ${\hat{Y}}_{1}(t)\left( Y_{2}(t)\right)$, and the other is using first $Y_{2}$ to estimate $Y_{3}$, and then estimate $Y_{1}$ from the last, i.e., ${\hat{Y}}_{1}(t)\left( {\hat{Y}}_{3}\left( Y_{2}(t)\right) \right)$, that is ${\hat{Y}}_{1}(t)={\hat{m}}_{1}\big ({\hat{Y}}_{3}(t)\big )$, where ${\hat{Y}}_{3}(t)={\hat{m}}_{3}\big (Y_{2}(t)\big )$. In the cases (i) and (ii) the trends of $Y_{1}$ estimated by ${\hat{Y}}_{1}(t)\Big (Y_{2}(t)\Big )$ and by ${\hat{Y}}_{1}(t)\Big ({\hat{m}}_{3}\big (Y_{2}(t)\big )\Big )$ are the same. In the case (iii) ${\hat{Y}}_{1}(t)\Big (Y_{2}(t)\Big )$ estimates an increase of $Y_{1}$ with the increase of $Y_{2}$, while ${\hat{Y}}_{1}(t)\Big ({\hat{m}}_{3}\big (Y_{2}(t)\big )\Big )$ estimates a decrease of $Y_{1}$ with the increase of $Y_{2}$. Therefore, observing the trend evolution of $Y_{2}$ does not clearly estimate the trend of $Y_{1}$ independently of the quality of the pairwise rank correlations. In other words, the trend of $Y_{1}$ is unpredictable from $Y_{2}$. A similar situation occurs for the case (iv).

**Figure 1:**
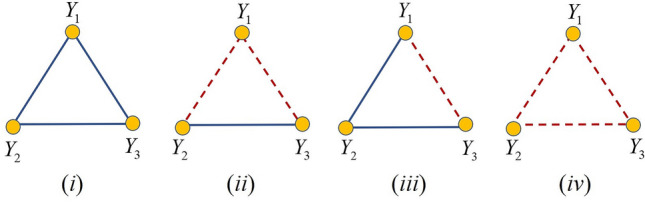
Graphical representation of the interdependencies between stocks $Y_{1}$, $Y_{2}$ and $Y_{3}$ in a triad. Stocks are represented as the vertices of a triangle and edges represent the correlation between them as accounted for by Kendall’s tau. Positive are represented as solid (blue) lines between the corresponding stocks, and negative are represented as dashed (red) lines.

In closing, the “predictability” of the trend of a stock $Y_{i}$ from that of $Y_{j}$ increases when the signs of the two estimations coincide. Otherwise, such predictability drops as a consequence of the different trends predicted by ${\hat{Y}}_{i}\left( t\right) \left( Y_{j}\left( t\right) \right)$ and ${\hat{Y}}_{i}(t)\Big ({\hat{m}}_{k}\big (Y_{j}(t)\big )\Big )$. If we represent the three stocks at the vertices of a triangle and the signs of the estimates as the corresponding edges, we have that the four cases analyzed before can be represented as in Fig. 1. Because the magnitude of $\tau _{ij}(t)$ quantifies the quality of the estimation for each *t*, we suggest to replace the edges of the triangles in Fig. 1 by the corresponding values of Kendall’s tau instead of their estimates signs (for more details see Appendix B in the SI). In this case, a measure of the predictability of the stocks in a given triad is given by ${\tilde{K}}(t)=\tau _{ij}(t)\tau _{ik}(t)\tau _{jk}(t)$, with ${\tilde{K}}(t)\rightarrow +1$ corresponding to larger predictability and with ${\tilde{K}}(t)\rightarrow -1$ to poorer predictability at time *t*. Obviously, we should extend this measure beyond triangles, which is what we do in the next Section.

A triangle like those illustrated in Fig. 1 is known as a signed triangle. A signed triangle for which the product of its edge signs is positive is known as a balanced triangle (cases (i) and (ii) in Fig. 1). Otherwise, it is said to be unbalanced (cases (iii) and (iv) in Fig. 1). These concepts are naturally extended to networks of any size by means of the concept of signed graphs (see next Section). A signed graph where all the triangles (more generally all its cycles) are balanced is said to be balanced. Therefore, we move our scenario of stock predictability to that of the analysis of balance in signed stock networks.

### Balance in weighted signed stock networks

Let the stocks in a given market be represented as a set of nodes *V* in a graph $G=\left( V,E,W\right)$, where $E\subseteq V\times V$ is a set of edges which represent the correlations between pairs of stocks, and $W:E\rightarrow [-1,+1]$ is a mapping that assigns a value between $-1$ and $+1$ to each edge. We call these graphs weighted signed stock networks (WSSNs). The edges of the WSSN are defined on the basis of time varying Kendall’s tau between the log returns of the stocks.

For any cycle in a WSSN we will say that it is positive if the product of all Kendall’s taus forming the edges of the cycle is positive. A WSSN is balanced if all its cycles are positive. However, the question is not to reduce the problem to a yes or not classification but to quantify how close or far a WSSN is from balance. To do so, we consider a hypothetical scenario in which a given WSSN $G=\left( V,E,W\right)$ has evolved from a balanced network $G'=\left( V,E,\left| W\right| \right)$. That is, we are interested in quantifying the departure of a given WSSN from a balanced version of itself. We define the equilibrium constant *K* for the equilibrium $G'\rightleftarrows G$ as $K=\exp \left( -\beta \triangle F\right)$, where $\beta$ denotes the inverse temperature of the network and $\triangle F$ is the change of the Gibbs free energy of the system (for more details about the components of the equilibrium constant see Ref.^41^). The equilibrium constant is bounded as $0<K\le 1$, with the lower bound indicating a large departure of the WSSN from its balanced analogous and reaching $K=1$ when it is balanced. We prove in Appendix C of the SI that this *K* is related to ${\tilde{K}}$, which was defined in the previous section to account for the product of the signs of all edges in the cycles of the WSSN.

To calculate balance in a signed network, Facchetti et al.^40^ used an intensive computational technique that assigns a $+1$ or a $-1$ to all the nodes so as to minimize the energy functional

2 $$\begin{aligned} h\left( \sigma \right) {:=}-\sum _{\left( v,w\right) \in E}J_{vw}\sigma _{v}\sigma _{w}, \end{aligned}$$

where $\sigma _{k}\in \left\{ +1,-1\right\}$, $k=1,\ldots ,n$ with *n* equal to the number of nodes and $J_{vw}$ accounts for the sign of the edge in the signed graph. Therefore, $h\left( \sigma \right) {:=}-\sum _{v,w}A_{vw}\sigma _{v}\sigma _{w}$, where $A_{vw}$ are the entries of the adjacency matrix of a signed graph. The energy functional $h\left( \sigma \right)$ is a quadratic form of the Hamiltonian: $h\left( \sigma \right) =\vec{1}^{T}{\hat{\mathscr {H}}}\left( G\right) \vec{1}$, where $\vec{1}$ is an all-ones vector. Here we use a different approach, which avoids the minimization of that energy functional, and which is based on the free energies $F\left( G\right)$ and $F\left( G'\right)$ appearing in $\triangle F=F\left( G\right) -F\left( G'\right)$. In this case we notice that the energy functional (2) is minimized if $J_{vw}\sigma _{v}\sigma _{w}>0$ and maximized if $J_{vw}\sigma _{v}\sigma _{w}<0$. Therefore, we identify this term with the Kendall’s tau: $J_{vw}\sigma _{v}\sigma _{w}{:=}\tau _{vw}$, which means that ${\hat{\mathscr {H}}}\left( G\right) =-A\left( G\right)$, such that correlated pairs of stocks contribute to the minimization of the energy functional and anticorrelated ones contribute in the opposite direction. Then, we have $\triangle F=-\beta ^{-1}\ln \left( Z\left( G\right) /Z\left( G'\right) \right)$, where $Z\left( G\right) =tr\left( \exp \left( -\beta {\hat{\mathscr {H}}}\left( G\right) \right) \right)$.

For the current work, we consider the Economic Policy Uncertainty (EPU) index^46^ as a proxy for the inverse temperature $\beta$. EPU is compiled for every country on a monthly basis and represents a level of “agitation” of the market at a given date. We then define a relative inverse temperature as $\beta _{rel}=EPU/\max \left( EPU\right)$, where $\max \left( EPU\right)$ is the maximum EPU reported for that country in the period of analysis. In this case, the risk is high when the “temperature” of the system at a given time is approaching the maximum temperature reached by that country in the whole period, $\beta _{rel}\rightarrow 1$. On the other hand, the risk is minimum when the temperature at a given time is much smaller than the maxim temperature of the period, $\beta _{rel}\rightarrow 0$.

This finally gives our measure of structural balance for a WSSN^41,47^:

3 $$\begin{aligned} K=\dfrac{Z\left( G\right) }{Z\left( G'\right) }=\dfrac{tr\left( \exp \left( -\beta _{rel}{\hat{\mathscr {H}}}\left( G\right) \right) \right) }{tr\left( \exp \left( -\beta _{rel}{\hat{\mathscr {H}}}\left( G'\right) \right) \right) }=\dfrac{tr\left( \exp \left( \beta _{rel}A\left( G\right) \right) \right) }{tr\left( \exp \left( \beta _{rel}A\left( G'\right) \right) \right) }. \end{aligned}$$

In the Supplementary Information (Appendix C) we prove that $K=1$ if and only if the WSSN is balanced. The departure of *K* from unity characterizes the degree of unbalance that the WSSN has.

### Weighted signed stock networks

In order to define the weight of the edges in the WSSNs we use Kendall’s tau rank correlation coefficients. Since we are interested in the time evolution of the stock networks, the edges are determined in terms of time varying rank correlations. The dependence between the stock log returns $(Y_{A},Y_{B})^{T}$ is estimated with the following nonparametric time varying Kendall’s tau estimator proposed by Ascorbebeitia et al.^45^:

4 $$\begin{aligned} {\hat{\tau }}_{A,B}\left( t\right) =\dfrac{4}{1-\sum _{s=1}^{S}w_{h}(t-s)^{2}}\sum _{s,r=1}^{S}w_{h}(t-s)w_{h}(t-r){\mathbb {I}}\left\{ Y_{A}(s)<Y_{A}(r),Y_{B}(s)<Y_{B}(r)\right\} -1 \end{aligned}$$

for $t=1,\ldots , S$, where $w_{h}(t-s)=(Sh)^{-1}k\left( \left( t-s\right) /\left( Sh\right) \right)$ is a sequence of kernel weights that smooths over the time space, $h>0$ is the bandwidth that regulates the degree of smoothness, and ${\mathbb {I}}\left\{ \cdot \right\}$ is the indicator function. In all our calculations we consider the Epanechnikov kernel $k\left( x\right) =\tfrac{3}{4}\left( 1-x^{2}\right) {\mathbb {I}}\left\{ \left| x\right| <1\right\}$. This kernel assigns a higher weight to those values of the indicator function that are close in time and lower weights to observations of the indicator farther away from *t*. The smoothing parameter *h* is selected minimizing the mean squared error of the Kendall’s tau estimator in (4) (for more details see Appendix A in the SI and Ref.^45^).

Let us now fix *t* and calculate ${\hat{\tau }}_{ij}\left( t\right)$ for every pair of stocks (*i*, *j*) in a given market and define the rank correlations matrix $M\left( t\right)$ whose entries are the values of ${\hat{\tau }}_{ij}\left( t\right)$. Let $\varepsilon \in {\mathbb {R}}^{+}$ be a given threshold. We create the matrix $A\left( t\right)$ whose entries are $A_{ij}\left( t\right) ={\hat{\tau }}_{i,j}\left( t\right) {\mathbb {I}}\{\left| {\hat{\tau }}_{i,j}\left( t\right) \right| \ge \varepsilon \}$. The threshold determines the value below which the correlation is considered to be negligible. Increasing $\varepsilon$ decreases the number of connections in the corresponding network. We have considered $\varepsilon =0.3$ a reasonable value. Moroever, the main findings are robust to alternative specifications, such as $\varepsilon =0.2$ and $\varepsilon =0.4$. The matrix $A\left( t\right)$ is then the adjacency matrix of a WSSN at time *t*. Here we construct weighted adjacency matrices based on daily returns from January 2005 until September 2020. Therefore, we have a set of WSSNs obtained with matrices $\mathscr {{\mathcal {A}}}=\left\{ A\left( t=1\right) ,\ldots ,A\left( t=S\right) \right\}$.

## Results

**Figure 2:**
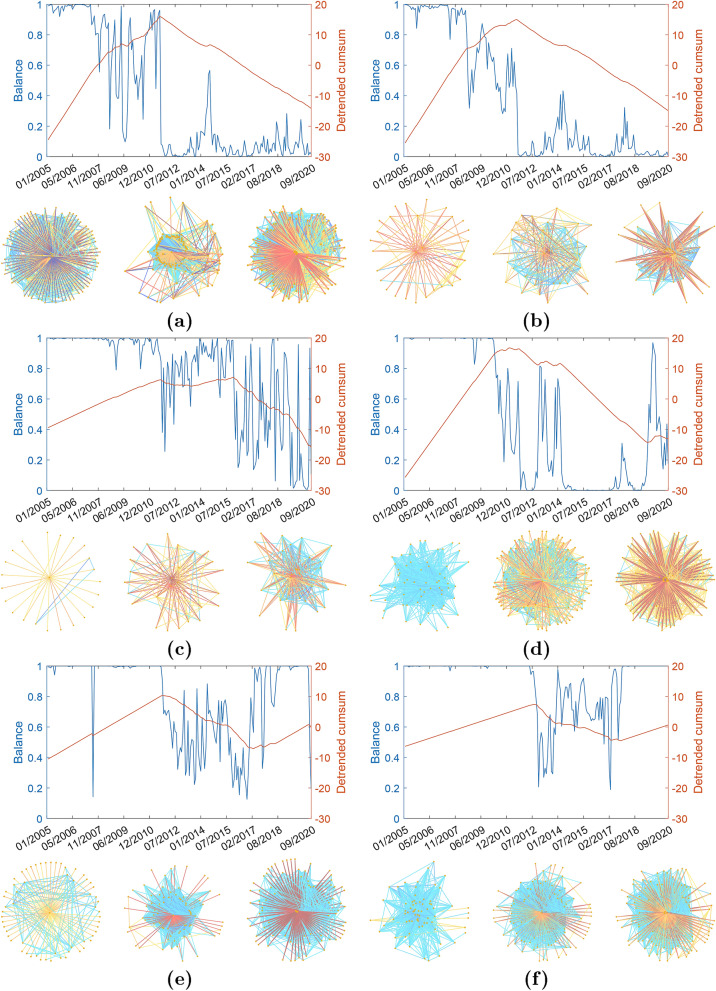
Balance degree evolution. (**a**) The US. (**b**) Portugal. (**c**) Ireland. (**d**) Greece. (**e**) Spain. (**f**) France. (Top panels) Illustration of the evolution of the balance for the period between January 2005 and September 2020 in the WSSN (blue line) and of its detrended cumulative sum (red line). (Bottom panels) Illustration of three snapshots of representative networks at different times (before, at, and after the BUT). The WSSNs are illustrated using the degree of the nodes as a proxy for the location of the nodes. The most central nodes are at the centre and the low degree nodes are located at the periphery of the graph. The colors of the edges correspond to the Kendall’s tau estimates, with links going from dark red for the most negative to blue for the positive ones. In the Supplementary Information we provide Matlab images of all the networks displayed in the figure where node labels are incorporated. The reader can zoom in these networks for details.

We study the stock markets of France, Germany, Greece, Italy, Ireland, Japan, Portugal, Spain, and the US during the period between January 2005 and September 2020 (see “Methods” section for details about data). The first interesting observation is the large balance observed for all markets between January 2005 and August 2007, when all of them display $K\ge 0.98$. However, in five countries there are sudden drops of balance around September/October 2011. This transition from highly balanced to poorly balanced markets occurs in Ireland in September 2011 and in the US, Portugal, Greece, and Spain in October 2011. The transitions are observed by naked eye in the plots of the temporal evolution of balance (see top panels of Fig. 2a–e). For a quantitative analysis of these BUTs as well as the identification of the dates at which they occurred, we use the detrended cumulative sums (DCS) of the balance (see Fig. 2). In these five markets, the general trend is a decay of the balance from 2005 to 2020. Therefore, the DCS increases for those periods in which the balance does not follow the general trend, i.e., when it does not decay with time. A negative slope of the DCS in some periods indicates that the balance drops more abruptly during this period than the general decreasing trend. Then, we can observe that DCS has a positive slope in all five markets with BUT between January 2005 and September/October 2011. At this point, the DCS changes its slope indicating an abrupt decay of balance. We select the point in which this change of slope occurs as the date marking the BUT. We should notice that there are some differences in the behavior of the DCS in each specific market. The US, Portugal and Greece display an initial increasing period followed by a continuous decay one. Ireland displays a short period in which DCS has slope slightly positive but close to zero between September 2011 and January 2016 when it definitively starts to decay. The market in Spain interrupted the decay of its balance on April 2017 when it starts to recover balance at a rate similar to the one of the period between January 2005 to October 2011. The value of the balance drops dramatically again after the crisis produced by COVID-19 as can be seen in Fig. 2e. Finally, we have included the stock market of France in Fig. 2f because it displays a behavior similar to that of Spain, although the BUT occurs significantly later with respect to the other countries in this group, i.e., on September 2012.

**Figure 3:**
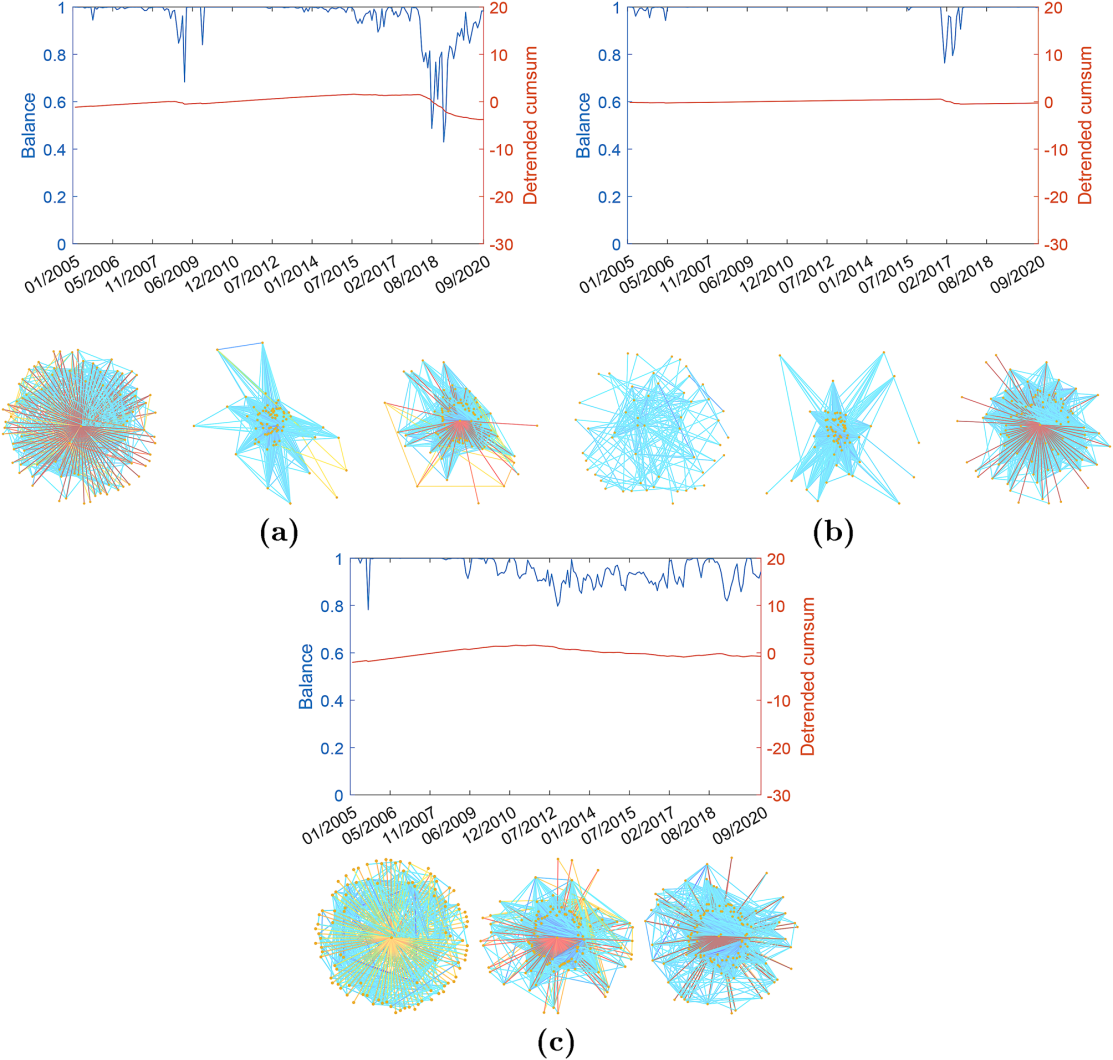
Balance degree evolution. (**a**) Germany. (**b**) Italy. (**c**) Japan. (Top panels) Illustration of the evolution of the balance for the period between January 2005 and September 2020 in the WSSN (blue line) and of its detrended cumulative sum (red line). (Bottom panels) Illustration of three snapshots of representative networks at different times (before, at, and after the BUT). The WSSNs are illustrated using the degree of the nodes as a proxy for the location of the nodes. The most central nodes are at the center and the low degree nodes are located at the periphery of the graph. The colors of the edges correspond to the Kendall’s tau estimates, with links going from dark red for the most negative to blue for the positive ones. In the Supplementary Information we provide Matlab images of all the networks displayed in the figure where node labels are incorporated. The reader can zoom in these networks for details.

The markets of Germany, Italy, and Japan display very constant values of their balance across the whole period of analysis. Although there are some oscillations at certain specific dates, their DCS display an almost zero slope confirming the constancy of the balance of these markets (see Fig. 3).

## Discussion

Among the factors that may influence the BUT in six out of nine countries studied, we should mention the global level of risk at which a given market is exposed to at a given time. We account for this factor through the parameter $0<\beta _{rel}\le 1$, which is based on the relative EPU index. Following our theoretical model, when the level of global risk is very low, $\beta _{rel}\rightarrow 0$, we have that $K\rightarrow 1$ and the corresponding WSSN tends to be balanced independently of its topology and edge weights. The analysis of the relative EPU values for all countries indicates that the level of risk accounted for by this indicator was relatively low from January 2005 to August 2007. This is exactly the same period for which a high balance was observed in all markets with $K\ge 0.97$. However, the relative EPU indices alone are not able to explain the BUTs observed in most markets around September/October 2011. For instance, the relative EPU index peaked at different values for different markets: Portugal (June 2016), the US (May 2020), Spain (October 2017), France (April 2017), Greece (November 2011) and Ireland (May 2020). It has been found that the US EPU can achieve better forecasting performance for foreign stock markets than the own national EPUs. This is particularly true during very high uncertainty episodes^48^ (see Appendix F in the SI for more details and references). Following this empirical observation, we can speculate that a plausible cause that triggered the BUT in five European countries were the events of August 2011, when the US EPU index rocketed to its highest value between 2005 and the COVID-19 crisis.

The topological analysis of BUT should necessarily start by considering the role of negative interdependencies. It is obvious that they are necessary for unbalance, i.e., a totally positive network cannot be unbalanced. However, it has been widely demonstrated in the mathematical literature that this is not a sufficient condition for unbalance, i.e., there are networks with many negative edges which are balanced^42,47^. As an illustrative example let us consider the WSSN of the US of September 3, 2010, which has 950 negative edges and a balance of $K\approx 0.245$ indicating its lack of balance. However, the US WSSN of February 18, 2011 also has 950 negative edges, but it is balanced with $K\approx 0.946$. Similarly, the WSSN of Portugal on July 6, 2012 ($K\approx 0.700$) and on May 3, 2019 ($K\approx 0.407$), both have 720 negative edges. The ratio of negative to total edges is neither a sufficient condition for balance. For instance, in Spain on August 12, 2005 the market was perfectly balanced $(K=1)$, while on September 2007 it was unbalanced ($K\approx 0.142)$, although both WSSNs have the same proportion of negative to total edges, i.e., 1.4%.

**Figure 4:**
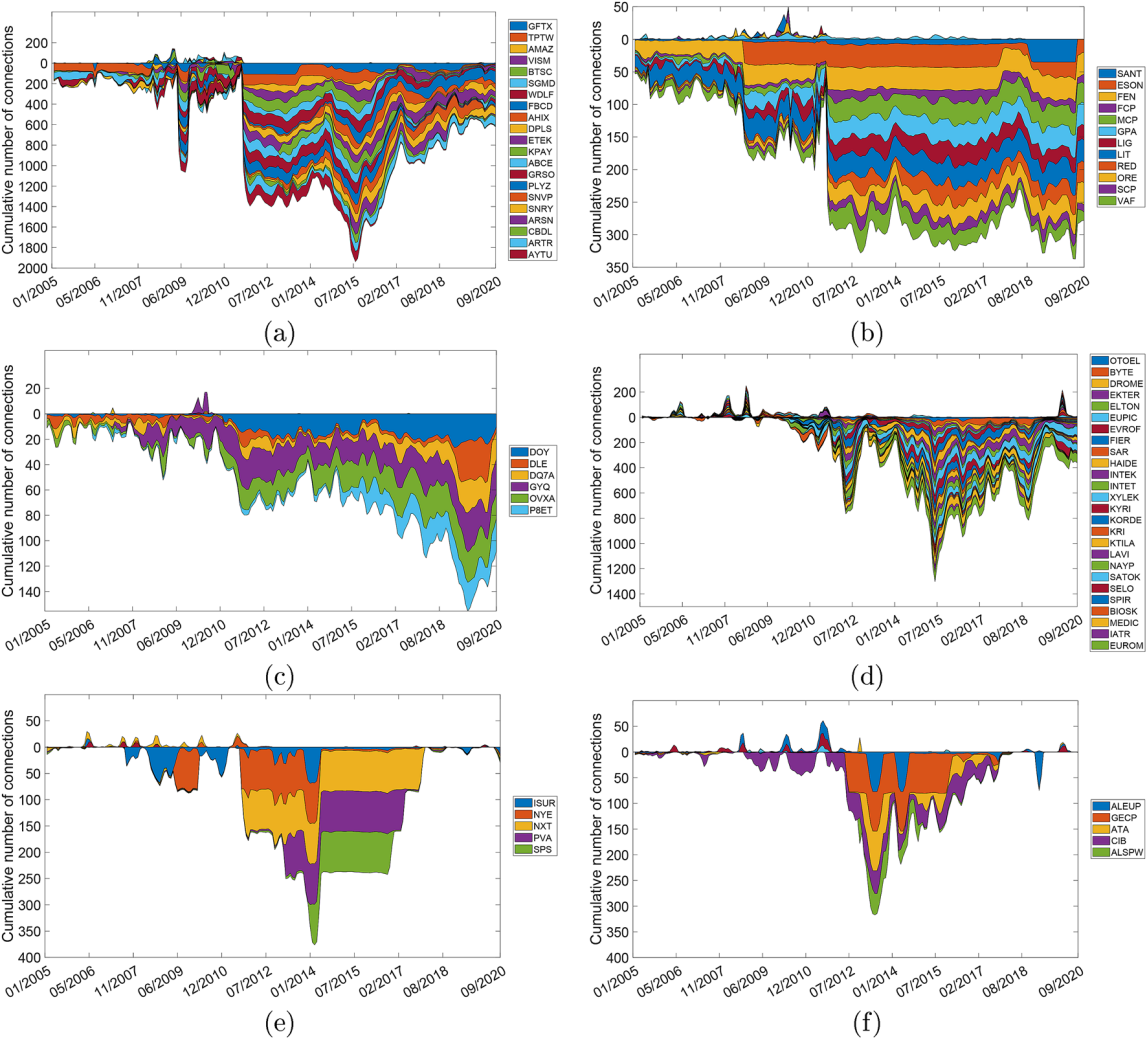
Positive and negative cumulative centrality degrees. (**a**) The US. (**b**) Portugal. (**c**) Ireland. (**d**) Greece. (**e**) Spain. (**f**) France. Temporal evolution of the cumulative number of connections (degrees) of every stock in the FNC of the six countries where BUT occurred. The cumulative number of positive connections with other stocks are shown over the horizontal line, and the cumulative number of negative connections are shown below that line.

A detailed exploration of the WSSNs with low balance occurring after BUT (see Fig. 2) revealed the existence of small fully-negative cliques (FNC) formed by a group of *s* stocks (see Appendix E in the SI for a greater evaluation of networks snapshots). A FNC is highly unbalanced. That is, the balance of an FNC of *s* stocks is $K\!=\!\left( \exp \left( -\beta _{rel}\left( s\!-\!1\right) \right) \!+\!\left( s\!-\!1\right) e^{\beta _{rel}}\!\right) \!/\!\big (\!\exp \left( \beta _{rel}\left( s\!-\!1\right) \right) \!+\!\left( s\!-\!1\right)$ $e^{-\beta _{rel}}\big )$, which for a fixed value of $\beta _{rel}$ clearly tends to zero as $s\rightarrow \infty$. Therefore, the unbalanced nature of the post-transition structure of stock markets is mainly due to the presence of these cliques of mutually anti-correlated stocks. Additionally, each of the stocks in the FNC increases significantly its number of negative connections with the rest of stocks after BUT (see Fig. 4), being also negatively connected to almost every other stock in the WSSN. This subgraph resembles a kind of graph known as *complete split graph* (CSG)^49^.

To explore the determinants of the lack of balance in these networks we simulate the structure of the networks displaying BUT by means of a quasi-CSG-WSSN (see “Methods” section for details) and compare it with a simulated random WSSN. The quasi-CSG-WSSNs have the same number of nodes and edges as the WSSNs but the size of the central clique is variable. We determine the “optimal” value of the sizes in the simulated networks, $s_{opt}$, by minimizing the root mean square error (RMSE) between the spectrum of the real WSSN and that of the quasi-CSG-WSSN. In SI Appendix G, Table S1, we report that the RSME of the random model is about twice bigger than that of the quasi-CSG for the six countries where BUT occurs. This clearly indicates that the topological organization, more than the number of positive/negative connections, is what determines the lack of balance in these networks.

The size of the FNC correlates very well with the intensity of the drop in the mean values of *K* before and after the BUT ($r\approx 0.857)$. Two other important characteristics of these FNC are that: (i) they are mainly formed by non-financial entities (see SI Appendix G, Table S1), and (ii) they are composed by small and micro-caps with the exception of SAR in Greece which is a mid-cap. We have shown (see Appendix H in the SI) that if we split the WSSN by financial (F) and non-financial (NF) sectors, then the BUT is observed mainly for the networks containing NF-NF interactions.

All in all, these BUTs change the predictability of the stocks in the way we have explained in “Correlations and predictability of interrelated stock prices” section from more predictable markets to more unpredictable ones.

## Conclusions

Considering stock-stock correlations in stock markets as signed graphs allowed us to introduce the concept of balance into stock market networks for the first time. We related the level of balance in these networks with stock predictability and identified a previously unknown transition between balanced markets to unbalanced ones in six out of nine countries studied. These balance-unbalance transitions occur in the US, Greece, Portugal, Spain, and Ireland around September 2011, following the Black Monday, and later on in France. No transition is observed in the stock markets of Germany, Italy, and Japan for the same period of time. The balance-unbalance transition is driven by a reorganization of the stock-stock correlations of a group of low capitalization stocks, mostly of non-financial entities which collapse into a fully-negative clique of anticorrelated pairs of stocks. Further studies are needed to understand the reasons and implications of this reorganization of non-financial entities in a given number of markets and the associated loss of network balance and stock predictability.

## Methods

### Data

We construct stock networks for some European countries, the US, and Japan. The seven selected European countries include Greece, Italy, Ireland, Portugal, and Spain (GIIPS), due to their financial vulnerability since the last global financial crisis. For comparison purpose, we have selected two other countries from the European Union: Germany and France. Finally, the US and Japan are included as non-European important drivers of the world economy. To construct the networks we use equities’ daily closing price data from 01-03-2005 to 09-15-2020, obtained from the Morningstar database. Details about the cleaning process of the data are given in the SI, Appendix D. This data set is available from the authors on reasonable request.

As the inverse temperature, $\beta$ of the networks we use monthly EPU index data^46^ for each country from January 2005 to September 2020. Portugal is an exception since there is no specific EPU index available for it. Therefore, the European EPU index is considered instead. EPU index data is publicly available in the Economic Policy Uncertainty website^50^.

### Quasi-CSG-WSSN

Let *n*, $m_{-}$ and $m_{+}$ be the number of nodes, of negative and positive edges in a real WSSN, respectively. We create a CSG with a clique of $s<n$ nodes and $s\left( 2n-s-1\right) <m_{-}$ edges. We complete the quasi-CSG-WSSN by adding randomly and independently $m_{-}-s\left( 2n-s-1\right)/2$ negative and $m_{+}$ positive edges among the nodes not in the clique. Additionally, we create the random-WSSN by adding randomly and independently $m_{-}$ and $m_{+}$ edges among *n* nodes a la Erdős-Rényi^51^. A Matlab code for building such graphs (Algorithm 1) and an example of quasi-CSG-WSSN are given in Appendix G of the SI.

We then test the following two hypotheses:

1. Unbalanced WSSNs depend only on *n*, $m_{-}$ and $m_{+}$ and not on any specific structure.
2. Unbalanced WSSNs depend on the existence of a specific quasi-CSG structure.

In the first case, the real-world WSSN would be more “similar” to its random version than to the quasi-CSG-WSSN and in the second case, it would be more similar to the quasi-CSG-WSSN. For the “similarity” between the different networks we focus here on the spectrum of their adjacency matrices. The reason for that is that the eigenvalues of this matrix determine the balance of the network as we have seen from its definition before. For determining the value of *s* we explore all possible values and find the root mean square error (RMSE) between the spectrum of the real WSSN and that of the quasi-CSG-WSSN. The smallest RMSE, $s_{opt}$, determines the “best” value of *s*.

## Supplementary information

Supplementary Information.
