# Author Correction: Loss of structural balance in stock markets

**DOI:** 10.1038/s41598-022-08518-0

**Published:** 2022-03-16

**Authors:** Eva Ferreira, Susan Orbe, Jone Ascorbebeitia, Brais Álvarez Pereira, Ernesto Estrada

**Affiliations:** 1 Department of Quantitative Methods, University of the Basque Country UPV/EHU, Avda. Lehendakari Aguirre 83, 48015 Bilbao, Spain; 2 Department of Economic Analysis, University of the Basque Country UPV/EHU, Avda. Lehendakari Aguirre 83, 48015 Bilbao, Spain; 3 Nova School of Business and Economics (Nova SBE) NOVAFRICA, and BELAB, Carcavelos, Portugal; 4 Institute of Mathematics and Applications, University of Zaragoza, Pedro Cerbuna 12, 50009 Zaragoza, Spain; 5 ARAID Foundation, Government of Aragon, Zaragoza, Spain; 6 Institute for Cross-Disciplinary Physics and Complex Systems (IFISC, UIB-CSIC) Campus Universitat de Les Illes Balears, 07122 Palma de Mallorca, Spain

Correction to: *Scientific Reports*, 10.1038/s41598-021-91266-4, published on 09 June 2021

The Funding section in the original version of this Article was omitted. The Funding section now reads:

“Brais Álvarez Pereira's work on this study was funded by Fundação para a Ciência e a Tecnologia (UIDB/00124/2020, UIDP/00124/2020 and Social Sciences Datalab – PINFRA/22209/2016), POR Lisboa and POR Norte (Social Sciences DataLab, PINFRA/22209/2016).”

The original Article has been corrected.

