# Supplementary material for: Loss of structural balance in stock markets
Source: Sci Rep. 2021 Jun 9;11:12230. doi: 10.1038/s41598-021-91266-4 (PMC8190088; doi:10.1038/s41598-021-91266-4)
Supplement: Supplementary file 1 — Supplementary Information. [file 41598_2021_91266_MOESM1_ESM.pdf]

Loss of structural balance in stock markets

Eva Ferreira[a], Susan Orbe[a], Jone Ascorbebeitia[b], Brais Álvarez Pereira[c], Ernesto Estrada[d,*]

[a]Department of Quantitative Methods, University of the Basque Country UPV/EHU, Avda. Lehendakari Aguirre 81, Bilbao, 48015 Spain

[b]Department of Economic Analysis, University of the Basque Country UPV/EHU, Avda. Lehendakari Aguirre 81, Bilbao, 48015 Spain

[c]Nova School of Business and Economics (Nova SBE), NOVAFRICA, and BELAB

[d]Institute of Mathematics and Applications, University of Zaragoza, Pedro Cerbuna 12, Zaragoza 50009, Spain; ARAID Foundation, Government of Aragon, Spain. Institute for Cross-Disciplinary Physics and Complex Systems (IFISC, UIB-CSIC), Campus Universitat de les Illes Balears E-07122, Palma de Mallorca, Spain.

[*]estrada66@unizar.es

A. Weighted Signed Stock Networks

For two stocks A and B in a given market, our interest is to investigate whether the relationship between the stock log returns $(Y_A, Y_B)^T$ changes with time. In this context, copulas are very useful since they give a flexible structure for modeling multivariate dependences [1]. Let F_A, F_B and, F be the continuous marginals and the joint distribution function of $(Y_A, Y_B)^T$. Then, based on Sklar's theorem [2], there is a unique copula function $C(\cdot,\cdot,t) : [0,1]^2 \rightarrow [0,1]$ such that $F(y_{At}, y_{Bt}, t) = C(F_A(y_{At}, t), F_B(y_{Bt}, t), t)$ for any $y_{jt} \in Y_j$, $j = A, B$. As a measure of the underlying dependence in the copula structure the Kendall's tau rank correlation is used, which can be written as a functional of the copula. Therefore, time varying Kendall's tau is defined in terms of copulas as

$$\tau_{A,B}(t) = 4 \int_{[0,1]^2} C(u_1, u_2, t) dC(u_1, u_2, t) - 1,$$

where $(u_1, u_2) \in [0,1]^2$. To estimate the time varying dependence, we use a nonparametric estimation method because of its advantage of overcoming the rigidity of parametric estimators. Specifically, it allows to remove the restriction that the joint distribution function

belongs to a parametric family. Therefore, Ascorbebeitia et al. [3] proposed to estimate the time varying copula $C(u_1, u_2, t)$ under α- mixing local stationary variables as

$$\hat{C}(u_1, u_2, t) = \frac{1}{Sh} \sum_{s=1}^{S} w_h(t-s) I\{Y_A(s) \leq \hat{F}_A^{-1}(u_1, t), Y_B(s) \leq \hat{F}_B^{-1}(u_2, t)\},$$

where $\hat{F}_j(y, t) = (Sh)^{-1} \sum_{s=1}^{S} w_h(t-s) I\{Y_j(s) \leq y\}$ denotes the nonparametric time varying estimator of the j-marginal distribution. In this context, they derive a consistent nonparametric estimator for the time varying Kendall's tau, $\hat{\tau}_{A,B}(t)$, which is defined in the "Methods" section of the main text.

B. Time Varying Nonparametric Regression

Let us consider the following regression model $Y_i(t) = m_i(Y_j(t)) + \epsilon_i(t)$, where $t = 1, ..., S$, $Y_i(t)$ is the dependent variable, $m_i(\cdot)$ is a non-specified unknown smooth function, $Y_{j \neq i}(t)$ is the explanatory variable and $\epsilon_i(t)$ is the error term. As pointed out in several studies (see, e.g., Refs. [4, 5, 6]), the time varying behavior of the variables is an important characteristic in finance to take into account. Since financial variables are dependent and not stationary, a nonparametric estimator for the regression model that accounts for time variation is considered under local stationary and α-mixing variables [7]. The time varying estimator of $m_i(y)$ for any value y in the domain of the variable Y_j is defined as

$$\hat{m}_i(y) = \left(\sum_{s=1}^{S} w_h(t-s) w_h(y - Y_j(s)) \right)^{-1} \sum_{s=1}^{S} w_h(t-s) w_h(y - Y_j(s)) Y_i(s),$$

where $w_h(y - Y_j(s)) = (Sh)^{-1} k\left((y - Y_j(s))/(Sh)\right)$ and $k(\cdot)$ denotes the kernel weights. To choose the smoothing parameter h, cross-validation methods proposed in the literature for nonparametric regression can be used (for details see, e.g., Ref. [8]).

If one is interested in the relation between regression slopes and correlation coefficients, a time varying relationship between variables can be assumed $m_i(Y_j(t)) = \beta_i(t) Y_j(t)$ (see Ref. [7]) leading to a semiparametric regression model. In such case, the time varying correlation between two variables, $\rho_{ij}(t)$, is related to the time varying slope $\beta_i(t)$ as $\rho_{ij}(t) = \beta_i(t) \left(\sigma_i(t)\right)^{-1} \sigma_j(t)$, where $\sigma_i^2(t)$ and $\sigma_j^2(t)$ are the time varying variances of variables Y_i and Y_j that can be estimated by smoothing the corresponding squared residuals. Then, $\hat{\rho}_{ij}(t)$

can be estimated through the time varying slope defined as

$$\hat{\beta}_i(t) = \left(\sum_{s=1}^{S} w_{h,ts} Y_j(s)^2\right)^{-1} \sum_{s=1}^{S} w_{h,ts} Y_i(s) Y_j(s)$$

In this setting, the estimation of $Y_i(t)$ is given by $\hat{Y}_i(t) = \hat{m}_i(Y_j(t)) = \hat{\beta}_i(t)Y_j(t)$. This relation would allow to replace the product of slopes of the regression models estimating the trend of the different pairs of stocks in a triad with the product of their respective conditional correlation and replace the study of predictability in stock markets by that of balance in WSSNs. Nevertheless, the linear correlation is not appropriate for financial variables as we have mentioned before. Hence, we consider the rank correlation as the connectivity measure between nodes in the network. Note that if the variables were gaussian, there is a one-to-one relationship between the linear correlation and Kendall's tau, i.e., $\rho_{ij}(t) = sin((\pi/2)\tau_{ij}(t))$, so using Kendall's tau generalizes the analysis made with linear correlation.

C. Balance

The definition of balance given in the main text is:

$$K = \frac{tr\left(\exp\left(\beta_{rel} A\left(G\right)\right)\right)}{tr\left(\exp\left(\beta_{rel} A\left(G'\right)\right)\right)},$$

which has also been defined in [9].

Let us first express the exponential of the adjacency matrix of the WSSN as a Taylor series (see for instance Ref. [10]):

$$tr\left(\exp\left(\beta_{rel} A\left(G\right)\right)\right) = n + \frac{\beta_{rel}^2}{2!} tr\left(A^2\left(G\right)\right) + \frac{\beta_{rel}^3}{3!} tr\left(A^3\left(G\right)\right) + \cdots .$$

Notice that $tr\left(I\right) = n$ and $tr\left(A\left(G\right)\right) = 0$. The term $tr\left(A^k\left(G\right)\right)$ represents the sum of all closed walks of length k in the WSSN. A *walk of length* k between the nodes v and u in an WSSN is the product of the Kendall's tau for all (not necessarily different) edges in the sequence $e_{v,1}, e_{1,2}, \ldots e_{k-1,u}$. The walk is closed if $v = u$. Then, $tr\left(A^2\left(G\right)\right) = \sum_{(i,j)\in E} \tau_{ij}^2$ and $tr\left(A^3\left(G\right)\right) = \sum_{(i,j,k)\in\triangle} \tau_{ij}\tau_{ik}\tau_{jk}$, where $\triangle$ is the triangle with vertices i, j, k. We can continue with higher order powers of $A\left(G\right)$, which are related to squares, pentagons, and so forth, apart from other cyclic and non-cyclic subgraphs. Obviously, we have already

shown here that for a signed triangle $tr\left(A^{3}\left(G\right)\right)=6\tilde{K}$, where $\tilde{K}=\tau_{ij}\tau_{ik}\tau_{jk}$ was defined in the main text of the paper as an index of predictability of the stock trends in a triad.

Let us first prove that $K=1$ if and only if the WSSN is balanced. For that, we first state a result proved by Acharya [11].

Theorem 1. *For any signed graph, the matrices $A\left(G\right)$ and $A\left(G'\right)$ are isospectral if and only if the signed graph is balanced.*

This means that both matrices $A\left(G\right)$ and $A\left(G'\right)$ have exactly the same eigenvalues $\lambda_{j}\left(A\left(G\right)\right)$ and $\lambda_{j}\left(A\left(G'\right)\right)$, respectively, if and only if the graph G is balanced. Then, we can write

$$K=\frac{\sum_{j=1}^{n}e^{\beta_{rel}\lambda_{j}(A(G))}}{\sum_{j=1}^{n}e^{\beta_{rel}\lambda_{j}(A(G'))}},$$

which is equal to one if and only if the graph is balanced.

Let us now show that K quantifies the departure of a WSSN from balance for non-balanced ones. Let us designate $W_{k}=tr\left(A^{k}\left(G\right)\right)$ the total number of CWs of length k in the WSSN. Obviously, $W_{k}=\sum_{i=1}^{n}W_{k}\left(i\right)$, where $W_{k}\left(i\right)$ is the number of CW of length k that starts (and ends) at the node i. Then, $W_{k}\left(i\right)<0$ if the node i is in an unbalanced cycle. Otherwise, $W_{k}\left(i\right)>0$. Therefore, $W_{k}\left(G\right)=\sum_{i=1}W_{k}^{+}\left(i\right)-\left|\sum_{i=1}W_{k}^{-}\left(i\right)\right|$, where $W_{k}^{+}\left(i\right)$and $W_{k}^{-}\left(i\right)$ are positive and negative CWs of length k starting at node i. Let us now designate $W^{+}\left(G\right)=\sum_{k=0}^{\infty}\sum_{i=1}\frac{\beta_{rel}^{k}}{k!}W_{k}^{+}\left(i\right)$ and $W^{-}\left(G\right)=\sum_{k=0}^{\infty}\sum_{i=1}\frac{\beta_{rel}^{k}}{k!}W_{k}^{-}\left(i\right)$. It is straighforward to realize that in G' $tr\left(\exp\left(\beta_{rel}A\left(G'\right)\right)\right)=W^{+}\left(G\right)+\left|W^{-}\left(G\right)\right|$, which implies that

$$K=\frac{W^{+}\left(G\right)-\left|W^{-}\left(G\right)\right|}{W^{+}\left(G\right)+\left|W^{-}\left(G\right)\right|}.$$

We can see now that $K=1$ only when the WSSN does not have any unbalanced cycle, i.e., $W^{-}\left(G\right)=0$. Also, K departs from one as the number of unbalanced walks growth, $K=1-\frac{2\left|W^{-}\left(G\right)\right|}{W^{+}\left(G\right)+\left|W^{-}\left(G\right)\right|}$, which approaches asymptotically to zero as $2\left|W^{-}\left(G\right)\right|\rightarrow\left(W^{+}\left(G\right)+\left|W^{-}\left(G\right)\right|\right)$.

D. Data

We use companies' daily data to construct stock networks for nine countries. The data set contains equities' daily closing price and volume data from 01-03-2005 to 09-15-2020.

The criteria used to consider a company as a candidate to form the database for each country is the trading volume at the end of April 2020. Initially, a set of around 250 companies with the highest trading volume at the 28th of April 2020 is considered, except for the US and Japan for which the initial sets are extended to 794 and 427 companies respectively because their stock markets are very diversified in terms of volume. Then, companies with a big amount of missing values (more than three market years of consecutive missing values or more than 30 % of non-consecutive missing values) according to the daily closing prices series have dropped out. There are many possible reasons for those missing values, such as trading cancellation for a period of time, stock market exit, late entry into the stock market, a merger of companies, bankruptcy, etc. We find that allowing for the 30% of missing values in a stock price series is a reasonable threshold. Nevertheless, there are some exceptions. Companies with more than 30% of missings but with a leading trading volume such as Abengoa S.A Class B (ABG.P) from the Spanish stock market, which constitutes the 39% of the total trading volume on the reference date, are excluded from this screening.

After the cleaning process, the database is composed of a set of representative companies according to the sector to which they belong (under Russell Global Sector classification) and their trading volume.

Table S1: Number of assets considered and the percentage of the total trading volume.

	Germany	France	Greece	Italy	Ireland	Portugal	Spain	US	Japan
no. of assets	81	78	73	83	32	36	78	119	120
volume	70.86%	76.9%	97.41%	90.56%	85.79%	98.75%	86.62%	45.66%	54.58%

Table S1 shows the number of companies considered and the percentage of the total trading volume that constitute such assets for each country.

E. WSSNs by country

Figures S1 to S3 illustrate the snapshots of three representative networks before, at, and after September 2011.

F. Events that may have triggered BUTs

In the paper, we have observed a lack of capacity of national EPU indexes to explain the balance transition in those countries that have experienced it. This might be due to the relationship between the US EPU index and foreign stock markets being stronger than that between these stock markets and their corresponding national EPU indexes. While there are some contradictory results depending on the chosen methodology [12], a large amount of evidence shows an association between the EPU index and greater stock price volatility for the US [13, 14, 15, 16, 17], with this association being particularly strong during very high uncertainty episodes [18]. However, the evidence for the relationship between national or European EPU indexes and stock market volatility for other countries is generally weaker [19], state dependent [20] and shows important levels of heterogeneity [12, 21]. Indeed, Mei et al. [22] find that models including the US EPU index can achieve better forecasting performance for European stock markets volatility, while the inclusion of the corresponding national own EPU index does not significantly increase forecasts accuracy. In this same direction, Ko and Lee [20] show that the negative link between the national EPU and stock prices changes over time, being importantly influenced by the co-movement of the national EPU index and the US one.

In the period considered in our study, the US EPU index attains its highest level before the COVID-19 crisis in August 2011, coinciding with the Black Monday. The Black Monday 2011 refers to August 8, when the US and the global stock market crashed, following the credit rating downgrade by Standard and Poor's of the US sovereign debt from AAA for the first time in history. This same period coincides with extremely high values for the US, European, and Asian stock market volatility indexes, VIX, VSTOXX, and VKOSPI respectively. These indexes - with an important leading role of the VIX on the other two through strong spillover effects- are established measures of fear, risk, and uncertainty in international stock markets, proved to be important in explaining stock returns [23]. The three of them attained their highest point between the 2008 financial crisis and the

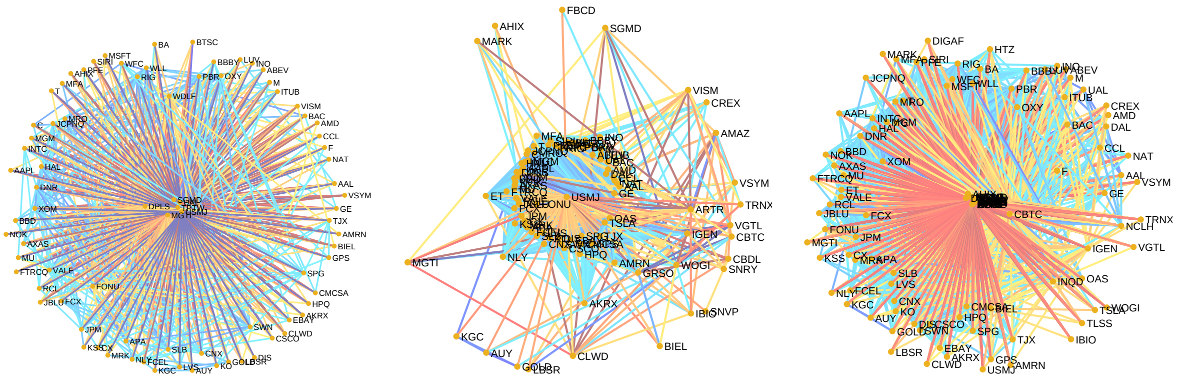

(a)

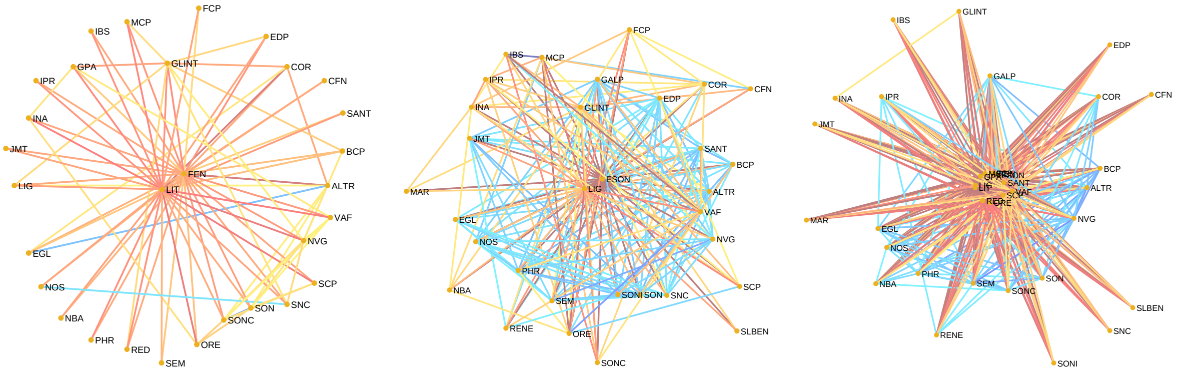

(b)

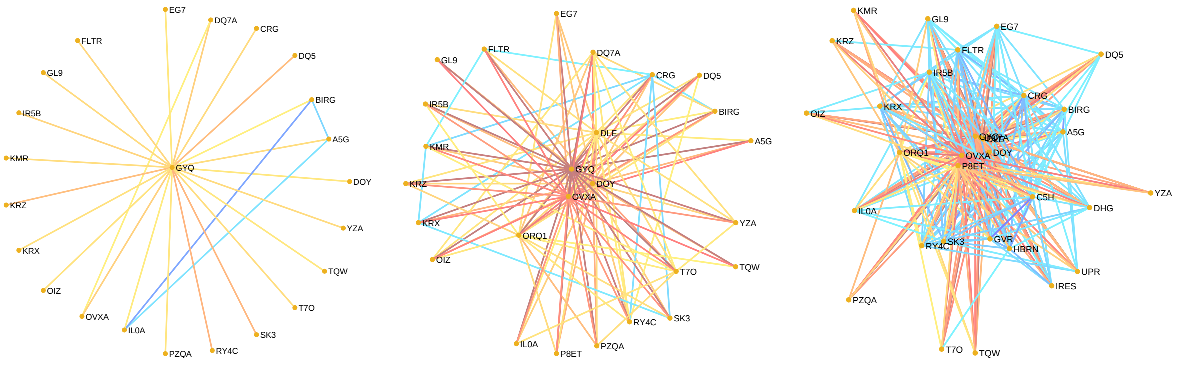

(c)

Figure S1: Snapshots for three representative networks before, at, and after the BUT. The colors of the edges go from dark red for the most negative Kendall's tau estimates to blue for the most positive ones. (a) The US. (b) Portugal. (c) Ireland.

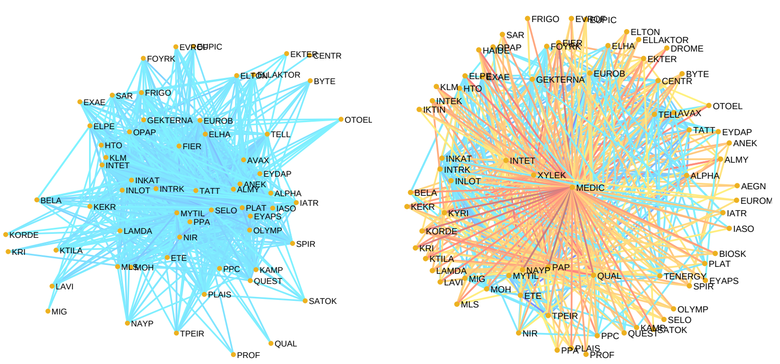

(d)

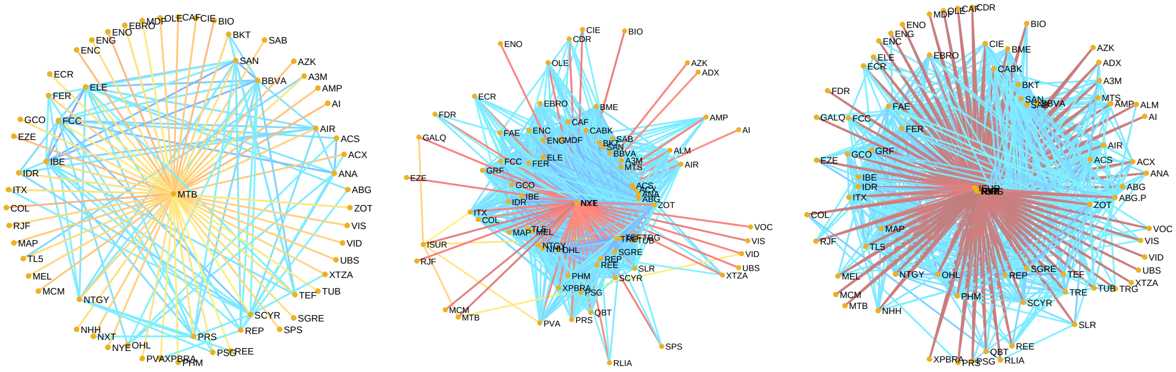

(e)

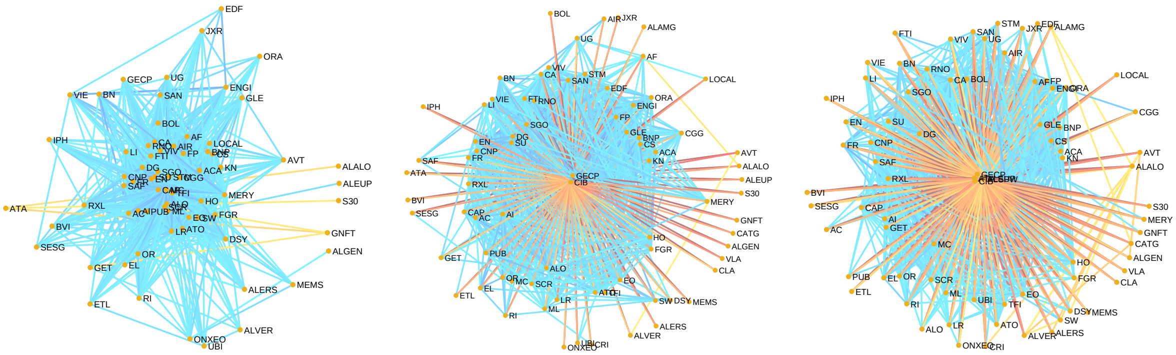

(f)

Figure S2: Snapshots for three representative networks before, at, and after the BUT. The colors of the edges go from dark red for the most negative Kendall's tau estimates to blue for the most positive ones. (d) Greece. (e) Spain. (f) France.

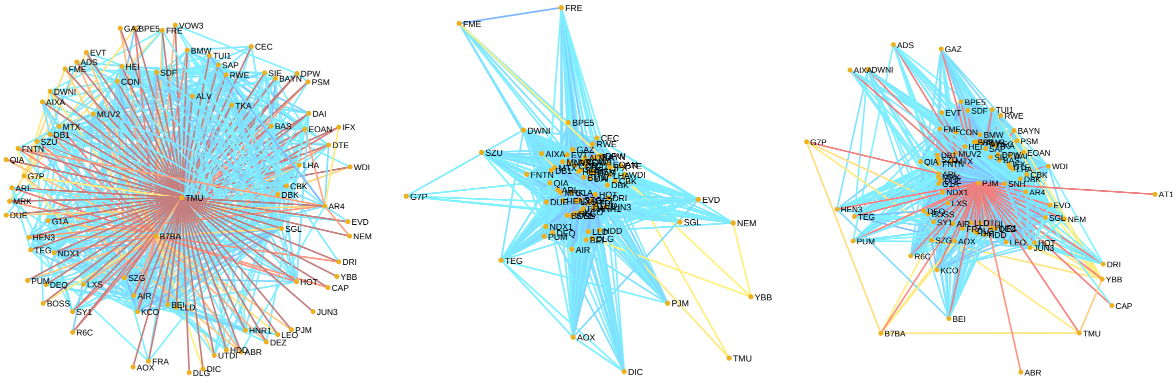

(g)

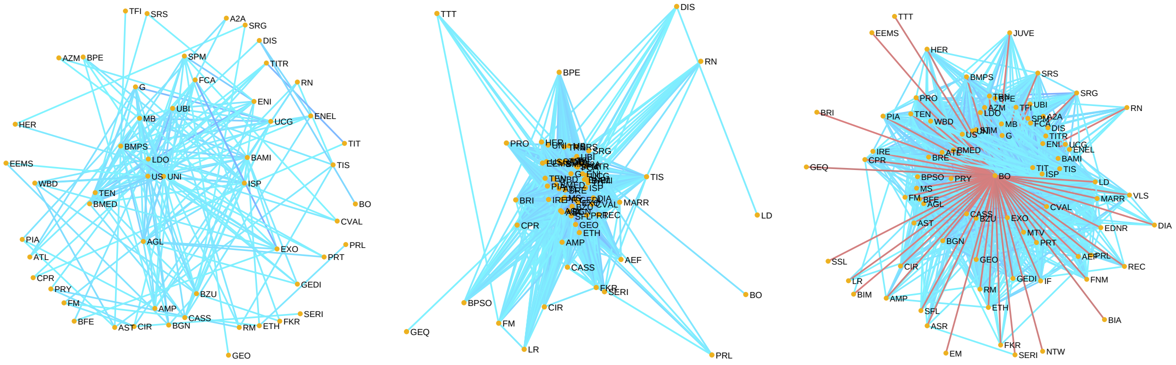

(h)

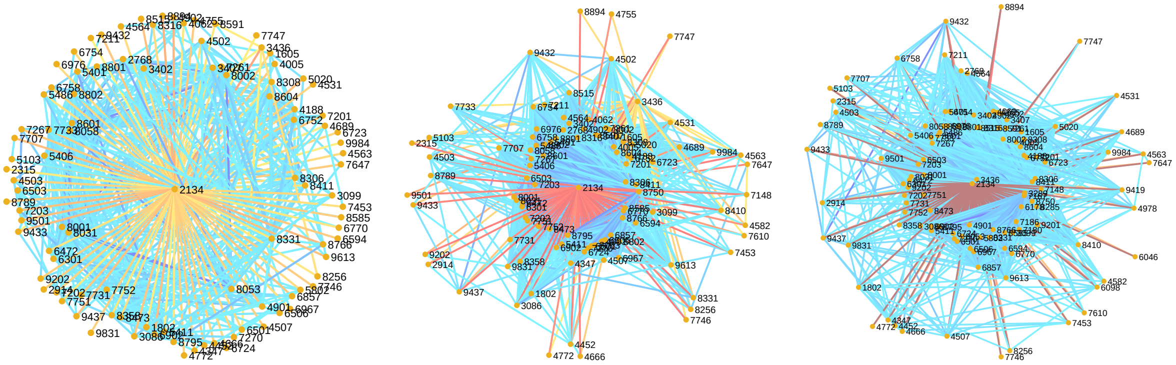

(i)

Figure S3: Snapshots for three representative networks before, at, and after the BUT. The colors of the edges go from dark red for the most negative Kendall's tau estimates to blue for the most positive ones. (g) Germany. (h) Italy. (i) Japan.

current COVID-19 crisis in September 2011, coinciding with the balance transition found in national stock markets.

These findings point towards the events associated with the Black Monday 2011 as the most likely triggers for the balance transition we observe in five of the countries in our sample. The association between a period of increased -economic policy- uncertainty and a sudden loss of balance seems coherent with the reduction of balance observed for most of the countries also after the crisis produced by COVID-19. However, this line of reasoning conflicts with the lack of a generalized, strong-enough drop in balance during the financial crisis of 2007-2008 in most of the countries. The US stock market did suffer significant balance fluctuations in this period, even if these were different from a strict loss of balance since balance was recovering after falling. A potential explanation might be the interruption of the negative time-varying correlation between policy uncertainty and stock market returns that took place during the financial crisis. This could be a consequence of the unprecedented bailout package for the US banking sector of 2008 and the stimulus package of 2009, which pushed the market into positive returns even if the policy uncertainty remained high [13].

A detailed exploration of the causes of the heterogeneous behavior of the different countries is out of the scope of this paper. Italy, Japan, and Germany, the three countries without a balance transition during the period had lower long-term GDP growth before the 2007-2008 financial crisis, but the obvious commonalities finish there. A promising avenue for the further exploration of this question might consider the differential impact of the European debt crisis in each country: the ranking of the countries according to the long-term interest rates of their national debt during this period matches the intensity of the observed balance transitions, except for Italy and France.

G. Quasi-CSG-WSSN

Algorithm 1 contains the Matlab code for building the quasi-CSG-WSSN.

In Figure S4 we illustrate an example of quasi-CSG-WSSN with $n = 50$, $m_- = 500$, $m_+ = 100$, and $s = 10$.

Table S2 contains the results for the analysis of the networks with BUT by means of

Algorithm 1 Matlab code for constructing a quasi-CSG-WSSN with given n, m_- and m_+ and s.

```
%%%Input data%%%
n=50;
m_neg=500;
m_pos=100;
s=10;

%%%Construction of the adjacency matrix A(G)%%%
B=-ones(n-s,s);
n_rest=n-s;
m_rest=m_neg-(s*(n-s)+s*(s-1)/2);
C=full(erdrey(n_rest,m_rest));
A=[-ones(s,s) B' B -C];
A=A-diag(diag(A));
A=triu(A);
K=tril(ones(n,n));
A=A+K+eye(n);
[row,col] = find(~A);
data=[row col];
y = datasample(data,m_pos,'Replace',false);
z=length(y);

for i=1:z
    A(y(i,1),y(i,2))=1;
end;

A=triu(A);
A=A+A';
A=A-diag(diag(A));
```

Table S2: Analysis of WSSNs with BUT. The number of companies s forming a central negative clique for each WSSN as well as the acronym for each company in such clique. The last three columns correspond to the analysis of the simulations of WSSNs with random and with quasi-CSG structures, where s_{opt} is the optimal value of s to minimize the root mean square error (RMSE) in the spectrum of the adjacency matrix of the quasi-CSG network relative to the real one. Underlined stocks correspond to the financial sector.

Market	Real WSSN		Simulated WSSN		
	s	Companies in clique	quasi-CSG		Random
			s_{opt}	RMSE	RMSE
Greece	26	BIOSK, BYTE, DROME, EKTER, ELTON, EUPIC, EUROM, EVROF, FIER, HAIDE, IATR, INTEK, INTET, KORDE, KRI, KTILA, KYRI, LAVI, MEDIC, NAYP, OTOEL, SAR, SATOK, SELO, SPIR, XYLEK	27	0.728	2.620
USA	21	ABCE, AHIX, AMAZ, ARSN, ARTR, AYTU, BTSC, CBDL, DPLS, ETEK, FBCD, GFTX, GRSO, KPAY, PLYZ, SGMD, SNRY, SNVP, TPTW, VISM, WDLF	21	1.810	4.516
Portugal	12	ESON, FCP, FEN, GPA, LIG, LIT, MCP, ORE, RED, SANT, SCP, VAF	12	1.095	2.465
Ireland	6	DEL, DOY, DQ7A, GYQ, OVXA, P8ET	6	1.131	2.255
Spain	5	ISUR, NXT, NYE, SPS, PVA	5	1.684	3.037
France	5	ALEUP, ALSPW, ATA, CIB, GECP	5	1.607	2.868

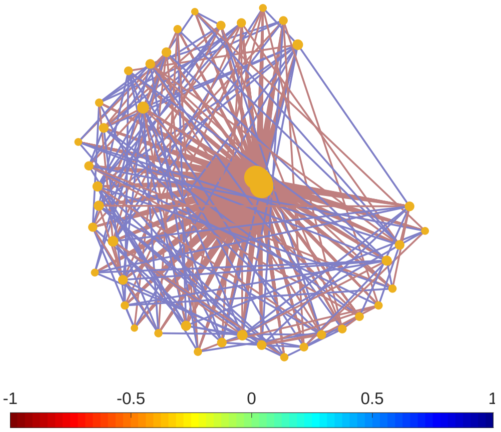

Figure S4: Illustration of a quasi-CSG-WSSN with $n = 50$, $m_- = 500$, $m_+ = 100$, and $s = 10$.

simulated quasi-CSG and random WSSNs.

H. WSSNs by financial and non-financial sectors

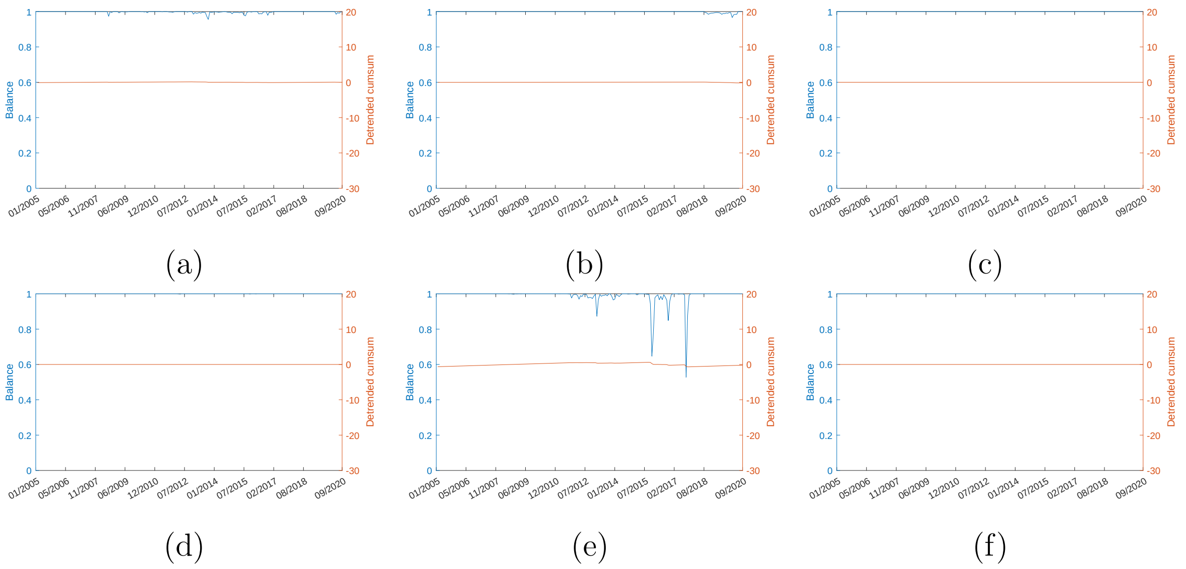

Figure S5: Evolution of the balance for stocks in the financial sector between January 2005 and September 2020 in the WSSN (blue line) and of its detrended cumulative sum (red line). (a) The US. (b) Portugal. (c) Ireland. (d) Greece. (e) Spain. (f) France.

Figures S5 and S6 show the evolution of the balance when we split the WSSNs by financial and non-financial sectors. Figure S7 presents the balance evolution for the WSSNs

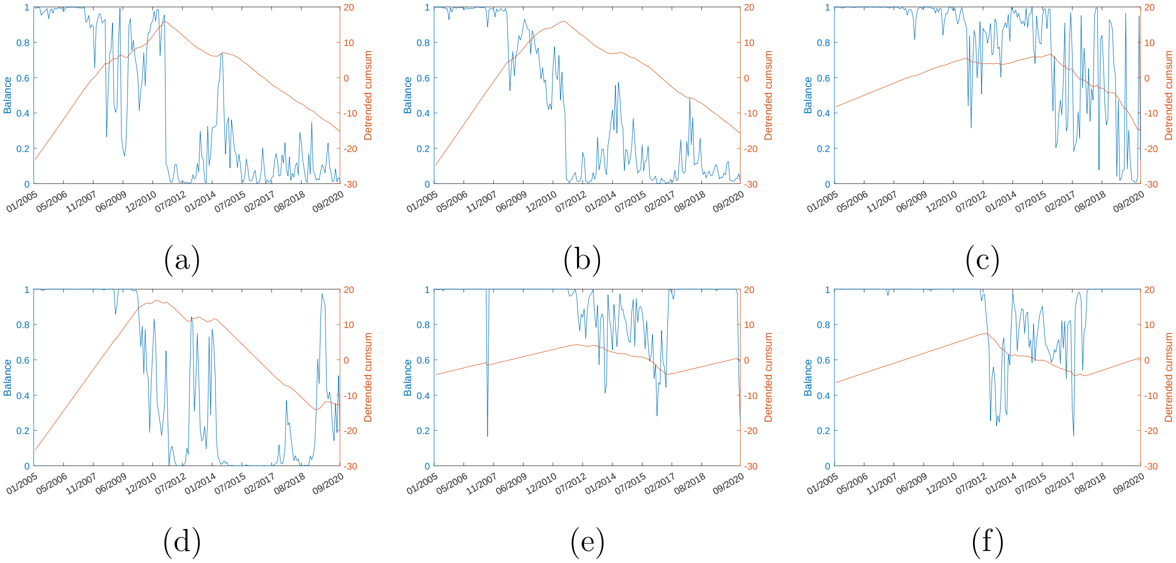

Figure S6: Evolution of the balance for stocks in the non-financial sector between January 2005 and September 2020 in the WSSN (blue line) and of its detrended cumulative sum (red line). (a) The US. (b) Portugal. (c) Ireland. (d) Greece. (e) Spain. (f) France.

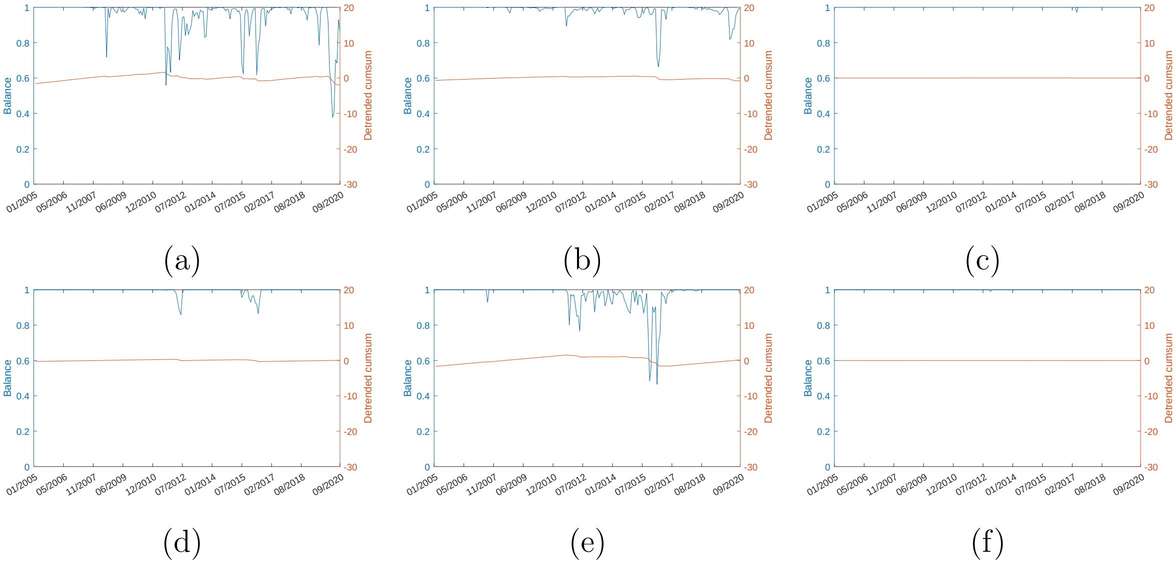

Figure S7: Evolution of the balance in the WSSN for interactions between financial and non-financial sectors' stocks from January 2005 until September 2020 (blue line), and of its detrended cumulative sum (red line). (a) The US. (b) Portugal. (c) Ireland. (d) Greece. (e) Spain. (f) France.

with cross interactions between financial and non-financial sectors' stocks.

References

[1] Roger B Nelsen. *An introduction to copulas.* Springer Science & Business Media, 2007.

[2] M Sklar. Fonctions de répartition à n dimensions et leurs marges. *Publications de l'Institut de Statistique de L'Université de Paris*, 8:229–231, 1959.

[3] Jone Ascorbebeitia Bilbatua, Eva Ferreira García, and Susan Orbe Mandaluniz. The effect of dependence on european market risk. A nonparametric time varying approach, 2021.

[4] C.N.V. Krishnan, Ralitsa Petkova, and Peter Ritchken. Correlation risk. *Journal of Empirical Finance*, 16:353–367, 2009.

[5] Eva Ferreira, Javier Gil-Bazo, and Susan Orbe. Conditional beta pricing models: A nonparametric approach. *J. Bank. Finance*, 35:3362–3382, 2011.

[6] Isabel Casas, Eva Ferreira, and Susan Orbe. Time-varying coefficient estimation in sure models. Application to portfolio management, 2020.

[7] Peter M. Robinson. Nonparametric estimation of time-varying parameters. In Peter Hackl, editor, *Statistical Analysis and Forecasting of Economic Structural Change*, pages 253–264. Springer Berlin Heidelberg, Berlin, Heidelberg, 1989.

[8] Jianqing Fan and Qiwei Yao. *Nonlinear time series: Nonparametric and parametric methods.* Springer Science & Business Media, 2008.

[9] Ernesto Estrada and Michele Benzi. Walk-based measure of balance in signed networks: Detecting lack of balance in social networks. *Physical Review E*, 90(4):042802, 2014.

[10] Ernesto Estrada. *The structure of complex networks: Theory and applications.* Oxford University Press, 2012.

[11] B Devadas Acharya. Spectral criterion for cycle balance in networks. *Journal of Graph Theory*, 4(1):1–11, 1980.

[12] Tsung-Pao Wu, Shu-Bing Liu, and Shun-Jen Hsueh. The causal relationship between economic policy uncertainty and stock market: A panel data analysis. *International Economic Journal*, 30(1):109–122, 2016.

[13] Nikolaos Antonakakis, Ioannis Chatziantoniou, and George Filis. Dynamic comovements of stock market returns, implied volatility and policy uncertainty. *Economics Letters*, 120(1):87–92, 2013.

[14] L'uboš Pástor and Pietro Veronesi. Political uncertainty and risk premia. *Journal of financial Economics*, 110(3):520–545, 2013.

[15] Li Liu and Tao Zhang. Economic policy uncertainty and stock market volatility. *Finance Research Letters*, 15:99–105, 2015.

[16] Jonathan Brogaard and Andrew Detzel. The asset-pricing implications of government economic policy uncertainty. *Management Science*, 61(1):3–18, 2015.

[17] Scott R Baker, Nicholas Bloom, and Steven J Davis. Measuring economic policy uncertainty. *Q. J. Econ.*, 131(4):1593–1636, 2016.

[18] Martin Bijsterbosch and Pierre Guérin. Characterizing very high uncertainty episodes. *Economics Letters*, 121(2):239–243, 2013.

[19] Xiao-lin Li, Mehmet Balcilar, Rangan Gupta, and Tsangyao Chang. The causal relationship between economic policy uncertainty and stock returns in china and india: Evidence from a bootstrap rolling window approach. *Emerging Markets Finance and Trade*, 52(3):674–689, 2016.

[20] Jun-Hyung Ko and Chang-Min Lee. International economic policy uncertainty and stock prices: Wavelet approach. *Economics Letters*, 134:118–122, 2015.

[21] Tihana Škrinjarić and Zrinka Orlović. Economic policy uncertainty and stock market spillovers: Case of selected cee markets. *Mathematics*, 8(7):1077, 2020.

[22] Dexiang Mei, Qing Zeng, Yaojie Zhang, and Wenjing Hou. Does us economic policy uncertainty matter for european stock markets volatility? *Physica A: Statistical Mechanics and its Applications*, 512:215–221, 2018.

[23] Hui-Chu Shu and Jung-Hsien Chang. Spillovers of volatility index: Evidence from us, european, and asian stock markets. *Applied Economics*, 51(19):2070–2083, 2019.
